# Numerical investigation of water inflow and seepage characteristics in a tunnel crossing two overlapped parallel faults

**DOI:** 10.1038/s41598-023-38986-x

**Published:** 2023-07-21

**Authors:** Jing Wu, Li Wu, Yan-hua Han, Miao Sun, Ya-ni Lu

**Affiliations:** 1 School of Civil Engineering & Hubei Town Development Research Center, Hubei Engineering University, Xiaogan, 432000 China; 2 Engineering Research Center of Rock-Soil Drilling & Excavation and Protection, Ministry of Education, China University of Geosciences, Wuhan, 430074 China

**Keywords:** Hydrology, Natural hazards, Environmental impact

## Abstract

Water inflow is one of the main geohazards that threaten the safety of tunnels and other underground engineering projects. Faulted zone is one of the important geological triggers for such events. Numerical investigations on the evolution of flow behavior in tunnels across fault zones are of significance to the predication and prevention of this type of geohazards. In this work, a numerical investigation model with two overlapped parallel faults is established at a steady stage according to the "Three Zones" fault structure theory. The rapid turbulent flow in the fault zone is simulated by using the improved Darcy-Brinkman seepage model, while the slow laminar flow in ordinary rock zone is described by Darcy equation. The effect of relative position and distance between the tunnel excavation face and overlapped parallel faults to the groundwater pore pressure and flow velocity is studied through several scenarios, and the water inflow rate into the tunnel is calculated. The numerical investigation results reveal that while the tunnel face is excavated into the fault center core, the fractured zone, the ordinary rock zone, and the center of the overlapped faults, the pore pressure value ahead of the excavation face increases while the flow velocity decreases sequentially. The inflow rate is the largest while the tunnel face is excavated to center of the fault center core, which is closely related to the range of the overlapped area. The investigation results offer a practical reference for predicting early warning of water inflow geohazard when a tunnel cross two overlapped parallel faults.

## Introduction

With the implementation of the "Belt and Road" initiative, infrastructure construction such as mining, transportation, water conservancy and hydropower projects in western China has made rapid progress^1^. At present, the depth, total length and total number of tunnels in China ranks the first place in the world^2–4^. Unfortunately, water and mud inrush has become a frequent and major geohazard during the deep-buried tunnel construction, causing substantial economic losses and casualties^5,6^. Avoiding water and mud inrush accidents caused by unfavorable geological formations such as faults and fractured rocks has become one of the focuses in underground engineering research^7–9^. Studying the evolution law of groundwater flow as a tunnel excavation face passes through fault zones is helpful to predict the inflow rate, and significantly reduce the water inrush risk, and avoid economic losses and casualties in tunnel construction^10^.

The evolution principles and manifestations of different water inflow channels and concluded that the development law of water inflow channels in fault zones is closely related to factors such as fluid pressure and blasting vibration^11^. Some scholars have put forward a multi-factor comprehensive investigation approach of water and mud inrush into tunnel under high temperature and high-pressure conditions and examined Based on the macro-geological model of large-scale fault zones^12,13^. Some scholars have conducted theoretical analyses, laboratory tests, and numerical simulations to understand the groundwater flow behavior and water inflow mechanisms of tunnels that cross faulted zones^14–16^. Theoretical analyses methods include the introduction of neural network, catastrophe theory, and other nonlinear approaches that describe the evolution of groundwater inflow. Zheng et al.^17^ proposed the conception of "activation coefficient" for filled karst tunnels and analyzed the evolution of unstable region over time. Laboratory tests include laboratory test, model test and field test et al. For example, Jeon and Wang et al.^18,19^ carried out physical model tests on wall rock stability and fracture development, analyzed the adverse influences of faults and poor bedding planes on stability and seepage characteristic of tunnel wall rock.

Prediction of the water inflow into tunnels, particularly in discontinuous rock masses, remains one of the most challenging issues faced in tunneling project, which can have devastating consequences and lead to obstacles in tunnel construction. There are various analytical, empirical, and numerical methods exist that can be used to determine the groundwater inflow rate into tunnels. Current estimating method of water inflow rate relies mostly on analytical solutions, however, there are no comprehensive and unique analytical method and the prediction accuracy of these methods is low, especially in fault zones with a high pervious feature^20^. It is difficult to calculate the water inflow rate in different space and construction time of the tunnel, so it is not suitable for the calculation and prediction of local water inflow of the tunnel^21^. Following classical damage mechanics and seepage mechanics, Tang et al.^22^ conducted experiments to understand the evolution of seepage during rock failure, established a numerical simulation model for water inflow induced by rock failure, and studied the evolution of rock failure caused by seepage. At the same time, numerical simulation is an effective means to analyze the mechanism of water gushing and mud outburst in tunnels, for example, elastic–plastic models and damage models based on FLAC and PFC have been applied to simulate the groundwater inflow process in tunneling with complex geological settings^23^ and high-pressure water-rich fault areas. Dynamic models for tunnel excavation have also been established by scholars in which excavation disturbance and fluid pressure are incorporated to simulate the development law of water inflow channels caused by the instability of surrounding rock in karst or fault tunnels^24–26^.

The reason why the geological disaster of water and mud inrush is difficult to contain when the tunnel passes through the complex fault zone is that the fluid–structure coupling mechanism in the process of surrounding rock instability and water inrush caused by tunnel excavation and unloading is still unclear. Due to complex engineering geological conditions and dynamic excavation conditions, it is important to learn the evolution law of water inrush into a tunnel under composite faults' impact, such as overlapped parallel faults. By using the "Three Zones" fault structure theory, two types of numerical models in the two parallel faults were established by solving the IDB seepage model to simulate the nonlinear underground water inflow into a tunnel. This work examined the development of pore pressure and flow velocity and analyzed how they influence water inflow rate into tunnel, at different distances between the tunnel face and the parallel faults.

## Methods

### Conceptual model

**Figure 1:**
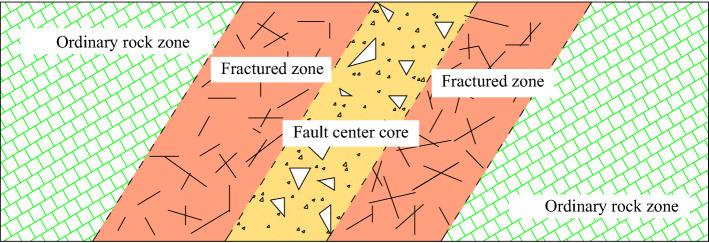
Schematic of the “Three Zones” fault structure.

A fault usually shows a certain strike and width, which indicating certain zoning characteristics. When the buried depth of the tunnel is fixed, the zonation of fault zone and the different characteristics of each zone play a decisive role in the stability of the surrounding rock and the water inrush disaster into tunnel. As shown in Fig. 1, when a tunnel is excavated crossing a single fault, it passes through five districts, that is the ordinary rock zone, the fractured zone, the fault center core, again the fractured zone, and the ordinary rock zone, which is the so-called "Three Zones" fault structure.

Specifically, in this “Three Zones” fault structure, the ordinary rock zones outside of the fractured zones has the same properties as the host rock which is not just influenced by the tectonic stress and the construction of tunnel, so it is not explained in detail here, but only the properties of fault center zone, the fractured zone are analyzed as following.

The fault center zone is the center zone of the fault. Due to the increased stress concentration, most of the energy and deformation in the geological tectonic movement of the fault are consumed. Various fault rocks and associated fractures develop in the fault center zone, and the rock mass is mainly cataclastic structure. The development of the associated fracturs that with different directions and with patterns will form the groundwater migration channel and cut the rock mass, increasing the permeability and water conductivity of the fault. In the fault center core, fractures often have high permeability because of no materials filling under normal conditions.

The fractured zone connects the fault center core and the ordinary rock zone as a transition. The distribution of cracks and weak planes spread in the fractured zone is affected by in situ stress, fault classification, lithology features, and the tunnel buried depth. Since cracks are usually not fully filled, they may also have high permeability. According to the development degree of fracture, it can be further subdivided into severe fault fractured zone, general fault fractured zone and slight fault fractured zone. From the severe fault fractured zone to the slight fault fractured zone, the distance from the fault center zone is increasing, the structural strength and fracture development density are decreasing, and the permeability gradually transitions to the ordinary surrounding rock characteristics.

### Numerical model

COMSOL Multiphysics (hereinafter referred to as COMSOL), with efficient computing performance and outstanding multi-field coupling analysis ability, can realize highly accurate numerical simulation, complete arbitrary multi-field, direct, two-way real-time coupling. Any independent function can be used to control the solving parameters. Material properties, boundary conditions and loads all support parameter control. It can solve the problems of flow- structure interaction and multi-flow coupling. In this work, the module import function of COMOSOL is used to import the deep-buried parallel composite fault tunnel model from CAD.

**Figure 2:**
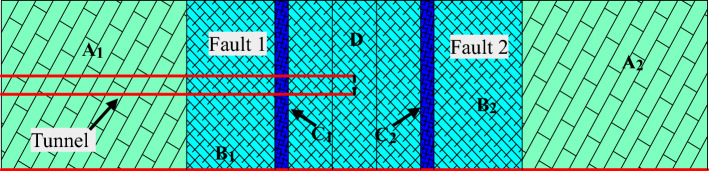
Numerical investigation model of a tunnel crossing two parallel faults. Zone A: the ordinary rock zone; zone B: the fractured zone of faults; zone C: the fault center core; zone D: the overlapped fault area.

The total length of Longjinxi diversion tunnel in Fujian province of China is 13.842 km. The greatest difficulty and danger is the massive frequently water inflow during the construction. According to the survey report and the field investigation situation, the water inflow disaster factor of the tunnel is faults, which doesn’t belong to the common karst tunnel water inrush. For example, the strike of fault F61 is perpendicular to that of the tunnel axis, with a dip angle of 42°, the widths of the core zone and damage zone on both sides are 3 m and 20 m, respectively. The base width of the tunnel face is 3 m and the upper semi-circular area is with a diameter of 3.9 m at the transverse section. The dimension of the simulation model is 160 m × 100 m × 100 m in the directions of X-axis, Y-axis, and Z-axis, with X-axis being the tunneling direction. The cross section of the tunnel is simplified into a circle with a diameter of 3.9 m. The tunnel burial depth is 350 m and the water level is 50 m below the surface. The two faults are parallel and perpendicular to the tunnel advancing direction, as shown in Fig. 2. Zone A is the ordinary rock zone, zone B is the fractured zone, zone C is the fault center core, and zone D is the overlapped fault area. The width of the fault center core is *d*_*1*_ = 3 m, the width of the fractured zone is *d*_*2*_ = 20 m, and the width of the overlapped zone is *d*_*3*_ = 10 m.

The fault center core and the fractured zone have a porosity of 0.5. Based on the parallel faults model, the impact of tunnel advancing step on the water inflow into tunnel is studied while crossing two parallel faults. Specifically, two parallel faults are designed with an overlapped damage zone, the tunneling process from entering the fault to passing through the fault is simulated. One of the most important parameters in the calculation of water inflow into tunnel is the permeability of surrounding rock. Assuming that the surrounding rock is a porous medium and the permeability of overlapped fault area conforms to superposition principle, the evolution of the seepage field at different working conditions is studied. Seven cases are designed in this study are provided in Table 1, in which *S* pertains to the distance from the tunnel excavation face to the center of overlapped parallel faults, and “-” indicates that the tunnel excavation face has not passed through the center of parallel overlapping fault, and vice versa.

**Table 1:** Simulation conditions for a tunnel crossing two parallel faults.

Parameter	Value						
Simulation conditions	*S* = −48	*S* = −28	*S* = *−*16.5	*S* = 0	*S* = *−*16.5	*S* = 28	*S* = 48

### Parameters and boundary conditions

In this work, on the premise of sufficient tunnel site investigation of the engineering geological and hydrogeological conditions, COMSOL is used to solve the Darcy equation for the slow laminar flow in the ordinary rock zone and the improved Brinkman equation for the fast nonlinear flow in the fault zone to mimic the groundwater flow behavior in the wall rock of tunnel crossing two overlapped parallel faults. The groundwater flow field was analyzed at a steady state.

Assuming that the ordinary rock zone is a porous medium, the slow laminar flow in the rock mass of ordinary rock zone satisfies a Darcy equation as,

1 $$\nabla \cdot \left( {\rho u} \right) = Q$$

2 $$V = - \left( {k/\mu } \right)\left( {\nabla P + \rho g\nabla D} \right)$$

where *V* is the flow velocity, $\rho$ is the fluid density, *Q* is the water inflow rate, *k* is the permeability of wall rock, *P* is the pore pressure, $\mu$ is the dynamic viscosity coefficient, and *D* is the parameter of position.

The ordinary rock zone has a pressure boundary condition, satisfying the following condition.

3 $$P = P_{0}$$

The value of *P* at the tunnel center axis (Z = 0) is set to 3 MPa according to the groundwater buried depth. The upper, back, left, and right boundary of this numerical model are influent boundaries, that is, the source recharge conditions of underground water flow. It is assumed that sufficient rainwater recharge conditions exist, and the inlet boundary is set as pressure boundary conditions, the pore pressure of the boundary meets the condition as *P*_*0*_ = *ρ*g(300-Z). Assuming that the tunnel wall within 1 m behind the working face has not taken support measures and is in contact with the atmosphere, the pore pressure at the working face and the tunnel wall 1 m behind is 0.

Except for the working face and the tunnel perimeter within 1 m behind, the supported tunnel wall on the tunnel perimeter, the bottom and front boundary of the model are all set as impermeable, which satisfying the following equation.

4 $$\left( {k/\mu } \right)\left( {\nabla P + \rho g\nabla D} \right) = 0$$

The flow velocity of the upper and back boundary, left and right boundary in the fractured zone and the fault center core can be expressed as:

5 $$\vec{n} \cdot \left\{ {\left( {1/\varepsilon_{p} } \right)\mu \left[ {\nabla V + \left( {\nabla V} \right)^{T} } \right]} \right\} = 0$$

Assume that* I* is the identity matrix, $\varepsilon_{p}$ is the porosity of wall rock, and *F* is the body force, the turbulent flow with high flow velocity in the fractured zone and fault center core can be solved by the Brinkman equation as below.

6 $$\left. \begin{aligned} \left( {{\eta \mathord{\left/ {\vphantom {\eta k}} \right. \kern-0pt} k}} \right)V & = \nabla \cdot \left\{ { - PI + \left( {{1 \mathord{\left/ {\vphantom {1 {\varepsilon_{p} }}} \right. \kern-0pt} {\varepsilon_{p} }}} \right)\left\{ \eta \right.} \right.\left. {\left[ {\nabla V + \left( {\nabla V} \right)^{T} } \right]} \right\}{\text{ + F}} \\ \nabla \cdot V & = 0 \\ \end{aligned} \right\}$$

Field water pressure test has been carried out in the vicinity of fault F61 of longjingxi diversion tunnel. The permeability variation law of the ordinary rock zone and fault zone is analyzed according to test data. According to the water pressure test results, the relationship between permeability *k* and distance *x* away from the center of the fault center core can be fitted by a Gaussian function. The permeabilities in the fault zone can be expressed by solving the improved Darcy-Brinkman seepage model, which is as follow by using the Gaussian equation.

7 $$\left\{ {\begin{array}{*{20}c} {k = k_{f} \cdot e^{{\frac{{\left( {\ln k_{f} - \ln k_{r} } \right)x^{2} }}{{\left( {\frac{{d_{1} }}{2} + d_{2} } \right)^{2} }}}} ,} & {\left| x \right| \le \frac{{d_{1} }}{2} + d_{2} } \\ {k = k_{f} ,} & {\left| x \right| \ge \frac{{d_{1} }}{2} + d_{2} } \\ \end{array} } \right.$$

where* k*_*f*_ is the value of the permeability in fault center core and in this work is* k*_*f*_ = 1 × 10^−11^ m^2^, *k*_*r*_ is constant value of the permeability in ordinary rock zone and in this work is* k*_*r*_ = 1 × 10^−16^ m^2^.

The groundwater flow follows Darcy equation in the ordinary rock zone, while follows the improved Brinkman equation in the fault fractured zone. The permeability Gaussian equation is achieved in the software COMSOL. In addition, based on the theories of mass conservation and pressure balance, *P* and *V* are continuous at the boundaries of the model as following.

8 $$P_{D} \left( {B_{{\text{i}}} } \right) = P_{B} \left( {B_{{\text{i}}} } \right),V_{D} \left( {B_{{\text{i}}} } \right) = V_{B} \left( {B_{{\text{i}}} } \right) \left( {{\text{i = }}1,2} \right)$$

By combining Eqs. (1) to (8), the Improved Darcy-Brinkman seepage model by obtaining the "Three Zones" fault structure theory has been conducted, with the abbreviation of “IDB seepage model” used in the following work.

## Results

### Excavated to 48 m behind the center of the parallel faults (S = −48)

(1) Numerical investigation results.

**Figure 3:**
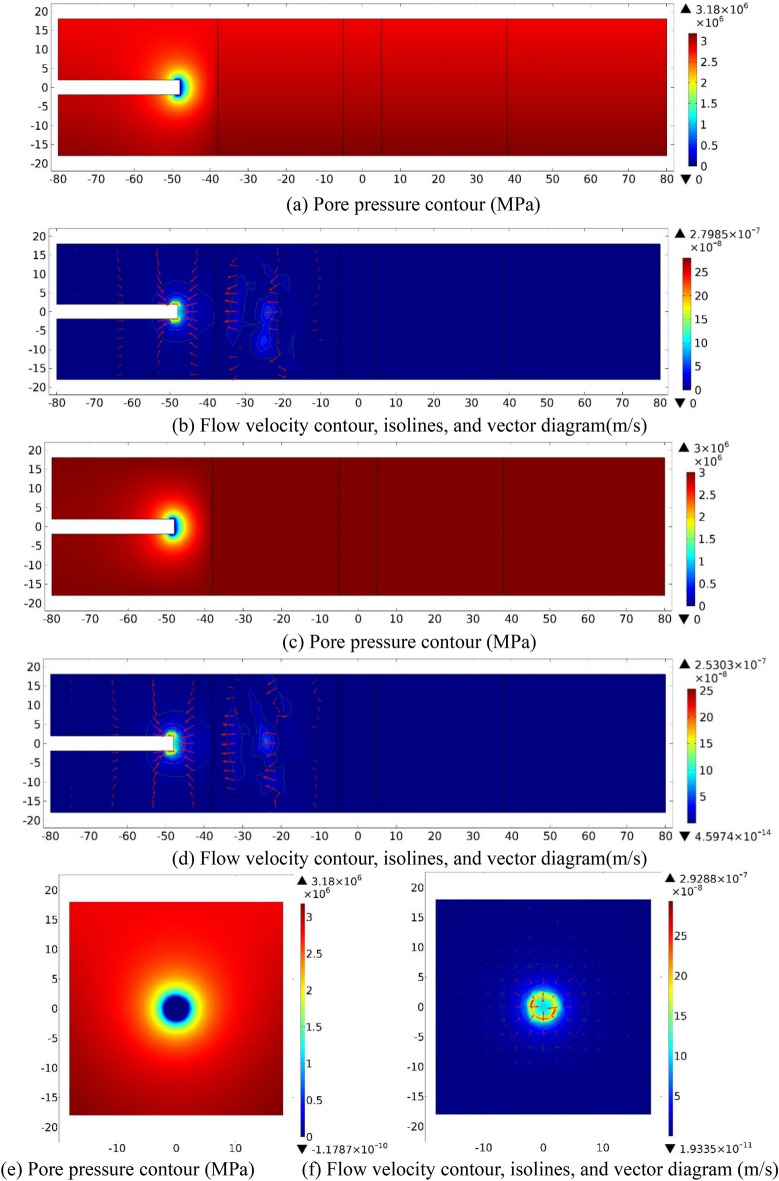
Numerical investigation results for the case *S* = −48 m. (**a**) and (**b**): XZ_Y=0_ section; (**c**) and (**d**): XY_Z=0_ section; (**e**) and (**f**): YZ_X=−48_ section.

While the tunnel face is excavated to 10 m away from the left boundary of the fractured zone of Fault 1(*S* = −48), the water inflow numerical investigation results on three sections (XZ_Y=0_, XY_Z=0_, YZ_X=−48_) are shown in Fig. 3 as follow.

As displayed in the *P* contours, pore pressure value (*P*) increases quickly from zero at the tunnel face to a certain value while *D* increases. On the section of XZ_Y=0_, *P* contours are approximately of an oval cross section, parallel to Z-axis on the long axis. Adjacent to the excavation face, the values of pore pressure gradient both in directions of the positive X-axis and the negative Z-axis is slightly greater than that in the positive Z-axis direction. On XY_Z=0_ section, *P* contours show a symmetrical distribution along X-axis, and the pore pressure gradient increases while the excavation face is closer to the overlapped faults. On the section of YZ_X=−48_, *P* contours also show a symmetrical distribution along Y-axis, and show a circular adjacent to the tunnel perimeter, then gradually display elliptical outwards, with the long axis parallel to the Y-axis. The value of *P* around the tunnel perimeter is small and it increases to a fixed value as the distance away from the tunnel increase. It can be seen from the velocity contours that the overall flow velocity within the model range is small, the maximum is about 10^−7^ m/s. The flow velocity decreases quickly outwards from a large value at and within 1 m behind the excavation face. On the sections of XZ_Y=0_ and XY_Z=0_, the groundwater flow fields at the vicinity of the working face are approximately symmetrical to the X-axis respectively, *V* contour shows an elliptical shape approximately, with the maximum value of 2.80 × 10^−7^ m/s and 2.53 × 10^−7^ m/s. On YZ_X=−48_ section, the flow velocity contour at the vicinity of to the tunnel perimeter is approximately circular, with a maximum value of 2.93 × 10^−7^ m/s. *P* contour at the vicinity of the excavation face it dissipates, forming a low permeability zone. The reason is that the tunnel working face is just passing through the ordinary rock zone.

(2) Evolution analysis of pore pressure and flow velocity

**Figure 4:**
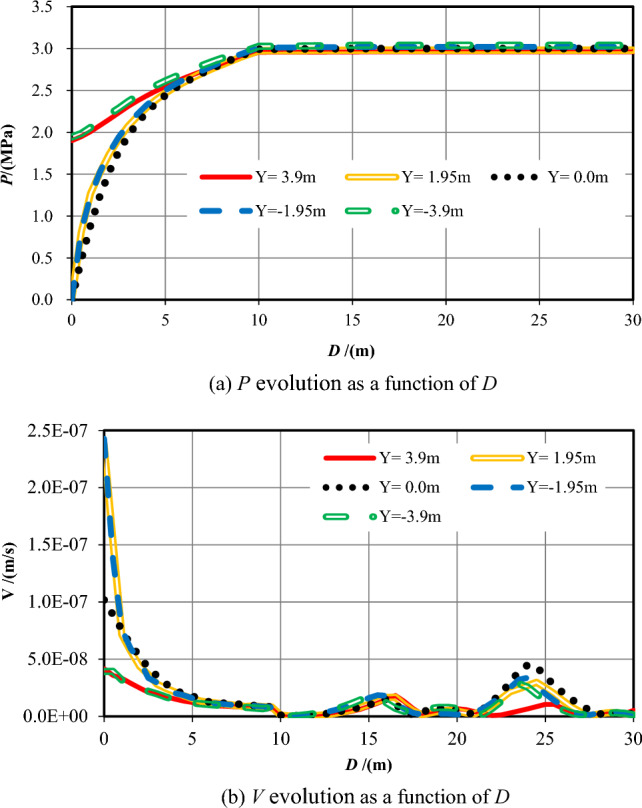
Evolution of pore pressure and flow velocity 30 m ahead of the excavation face (*S* = −48 m).

Table 2 reveals the information of the survey range and the measuring points number while S = −48. Figure 4 shows the evolution data of *P* and *V* that monitored within 30 m ahead of the excavation face as a function of distance away from the tunnel face (*D*).

**Table 2:** Information of the measuring points while *S* = *−*48.

No. of measuring line	Survey range	Number
1	X = *−*48–2 m, Y = 3.90 m	100
2	X = *−*48–2 m, Y = 1.95 m	100
3	X = *−*48–2 m, Y = 0.00 m	100
4	X = *−*48–2 m, Y = *−*1.95 m	100
5	X = *−*48–2 m, Y = *−*3.90 m	100

Figure 4a reveals that while Y =  ± 3.9 m, the pore pressures are similar because of the equal distance from the excavation face, which gradually increase from about 1.90 MPa approximately at the working face to 3 MPa as *D* increases (0 < *D* ≤ 10 m). While Y =  ± 1.95 m and 0, the pore pressure increases first rapidly and then slowly from 0 at the working face within 10 m in front, eventually stabilized at around 3 MPa. While 0 < *D* ≤ 5 m, pore pressures at Y =  ± 3.9 m are greater than that at Y =  ± 1.95 m and 0. While 5 m ≤ *D* ≤ 10 m, pore pressures are almost the same. Overall, when *S* = −48 m, *P* at the working face is small and increases quickly and then slowly as *D* further increases.

Figure 4b reveals that while Y =  ± 3.9 m, *V* declines quickly ahead (0 < *D* ≤ 5 m) and then slowly deep into the formation of the wall rock (5 < *D* ≤ 10 m), afterwards, it approaches to 0. The maximum value of *V* (*V*_*max*_) appears at the excavation face, that equals to 4.17 × 10^−8^ m/s when Y =  + 3.9 m and equals to 3.93 × 10^−8^ m/s when Y = −3.9 m, respectively. While Y = 1.95 m, 0 and −1.95 m, *V*_*max*_ is 2.37 × 10^−7^ m/s, 1.02 × 10^−7^ m/s, and 2.43 × 10^−7^ m/s respectively at the tunnel face. Flow velocity decreases quickly ahead (0 < *D* ≤ 1 m) and then declines slowly (1 < *D* ≤ 5 m), afterwards, it keeps decreasing to 0 (5 < *D* ≤ 10 m). While 0 < *D* ≤ 5 m, the values of *V* while Y = 1.95 m, 0 and −1.95 m are much bigger than that of Y =  ± 3.9 m. While *D* > 5 m into the formation, the value of *V* increases with increasing Y generally. In general, the value of *V* is big within a small distance ahead, but it declines quickly and then tardily as the distance further increases. All flow velocities fluctuate up and down adjacent to *D* = 16 m and *D* = 24 m. Down the Y-axis, the value of *V* deeper into the foundation is much smaller than that at the tunnel section.

### Excavated to 28 m behind the center of the parallel faults (S = −28)

(1) Numerical investigation results.

**Figure 5:**
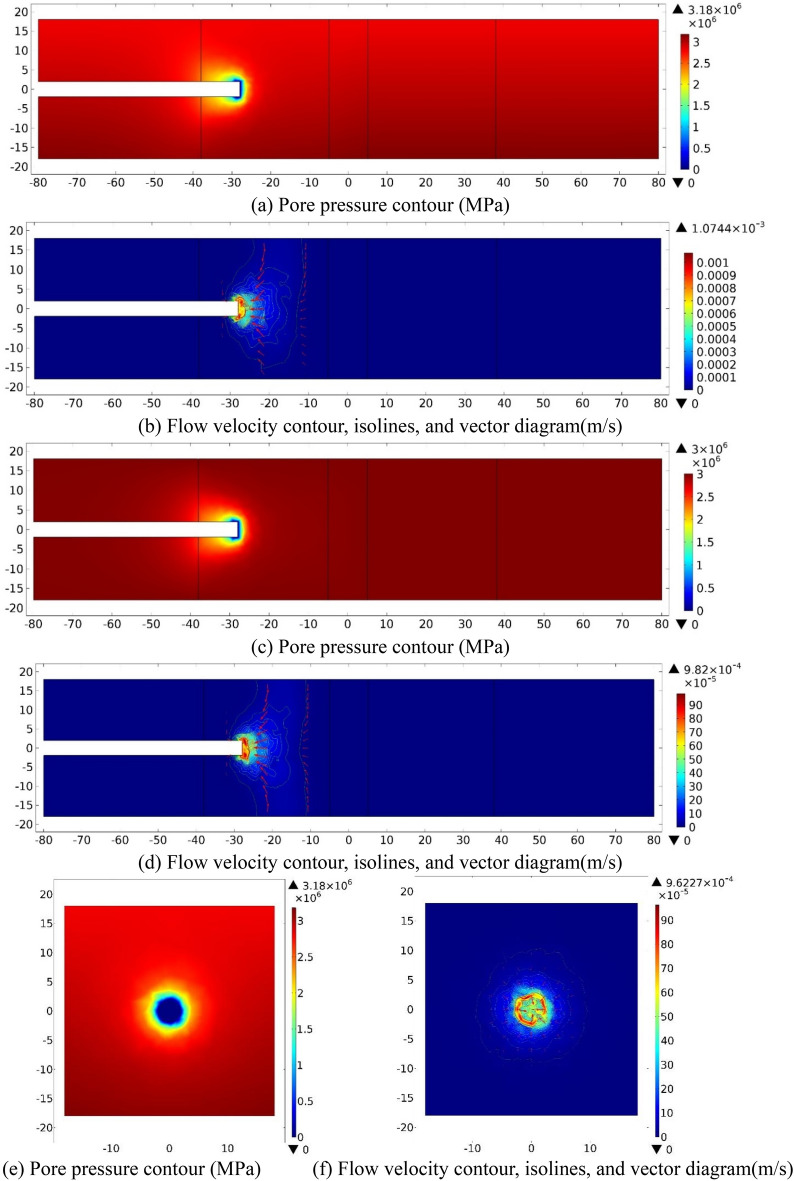
Numerical investigation results for the case *S* = −28 m. (**a**) and (**b**). XZ_Y=0_ section; (**c**) and (**d**): XY_Z=0_ section; (**e**) and (**f**): YZ_X=−28_ section.

When tunnel face is excavated to the center of the fractured zone of Fault 1 (S = −28), water inflow numerical investigation results on three sections (XZ_Y=0_, XY_Z=0_, YZ_X=−28_) are shown in Fig. 5 as follow.

As plotted in Fig. 5a,c,e, while the tunnel face is excavated to the center of the left fractured zone of Fault 1 (*S* = −28 m), the *P* value at the excavation face and around the tunnel perimeter is 0, which gradually increases outwards and reaches to a fixed value. On the XZ_Y=0_ section, at the vicinity of the tunnel excavation face, the pore pressure gradient in the positive direction of X-axis is slightly greater than that in Z-axis. The gradient behind the excavation face is small, then increases to a fixed value with the burial depth from the excavation face. On the section of XY_Z=0_, *P* contour presents an oval distribution with the major axis parallel to X-axis. At the vicinity of the excavation face, the pressure gradient on the X-axis is slightly greater than that on the Y-axis. On YZ_X=−28 m_ section, pressure distribution changes step by step from a circular distribution outward to an approximately oval cross section, and the long axis is parallel to Y-axis. Then the pore pressure value increasing to reach a fixed value, finally.

As plotted in Fig. 5b,d,f, the velocity contours, isolines, and vector diagrams show that when *S* = −48, the value of *V* at the tunnel face and within 1 m range of is significant, while groundwater flows towards the tunnel excavation face. On XZ_Y=0_ section, the flow velocity contour presents a circular arc distribution and gradually offsets to the positive Z-axis, with a maximum of 1.07 × 10^−3^ m/s. On XY_Z=0_ section, the flow velocity contour presents an oval distribution about the X-axis, the velocity contour diffuses outward in a circular arc distribution, and the maximum value of *v* is 9.82 × 10^−4^ m/s. On YZ_X=−28_ section, the flow velocity contour around the circumference of the tunnel is approximately circular distribution, with a maximum of 9.62 × 10^−4^ m/s. A low pressure area is presented at and around the tunnel perimeter that 1 m behind the excavation face, while groundwater flows toward the tunnel excavation face.

(2) Analysis and summary

**Figure 6:**
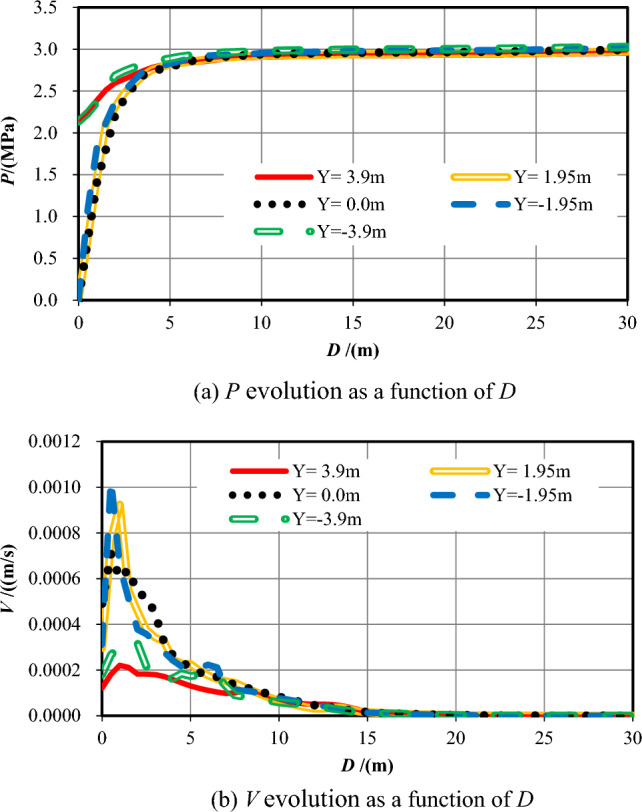
Pore pressure and flow velocity 30 m ahead of the excavation face (*S* = −28).

Table 3 reveals the information of the survey range and the measuring points number while *S* = −28. Figure 6 shows the evolution data of *P* and *V* that monitored within 30 m ahead of the excavation face as a function of *D*.

**Table 3:** Information of the measuring points while *S* = *−*28.

No. of measuring line	Survey range	Number
6	X = *−*28–22 m, Y = 3.90 m	100
7	X = *−*28–22 m, Y = 1.95 m	100
8	X = *−*28–22 m, Y = 0.00 m	100
9	X = *−*28–22 m, Y = *−*1.95 m	100
10	X = *−*28–22 m, Y = *−*3.90 m	100

Figure 6a plotted that while Y =  ± 3.9 m, *P* contours are similar because of the equal distance from the excavation face, that gradually increase from 2.10 MPa approximately at the working face to 3 MPa as *D* increases (0 < *D* ≤ 5 m). While Y =  ± 1.95 m and 0, the pore pressures increases first rapidly and then slowly from 0 at the working face within 5 m in front, eventually stabilizing at around 3 MPa. While 0 < *D* ≤ 5 m, pore pressures when Y =  ± 3.9 m are greater than that when Y =  ± 1.95 m and 0. While 5 m ≤ *D* ≤ 10 m, the evolution law of the pore pressures are almost the same. Overall, when *S* = −48 m, *P* on the working face is small and increases quickly and then slowly as the distance further increases. While 0 < *D* ≤ 1.5 m, the pore pressure value when Y = 3.9 m is slightly bigger than that when Y = −3.9 m.

Figure 6b reveals that while Y =  ± 3.9 m, *V* declines quickly ahead (0 < *D* ≤ 1.5 m) and then declines slowly into the formation (1.5 < *D* ≤ 7 m), afterwards, it changes slightly approaching to 0. The maximum values (*V*_*max*_) appear nearby the tunnel face when D = 1 m and D = 1.5 m, which are 2.21 × 10^−4^ m/s m/s at Y =  + 3.9 m and 3.04 × 10^−4^ m/s at Y = −3.9 m, respectively. While Y = 1.95 m, 0, and −1.95 m, *V* increases quickly ahead (0 < *D* ≤ 1 m), and then declines slowly (1 < *D* ≤ 6 m), afterwards, it keeps decreasing to 0 (6 < *D* ≤ 10 m). while 0 < *D* ≤ 7 m, the values of *V* while Y =  ± 1.95 m and 0 are much bigger than that of Y =  ± 3.9 m. While *D* > 7 m, the values of *V* are nearly equal and gradually approach 0.

### Excavated to 16.5 m behind the center of the parallel faults (S = −16.5)

(1) Numerical investigation results

**Figure 7:**
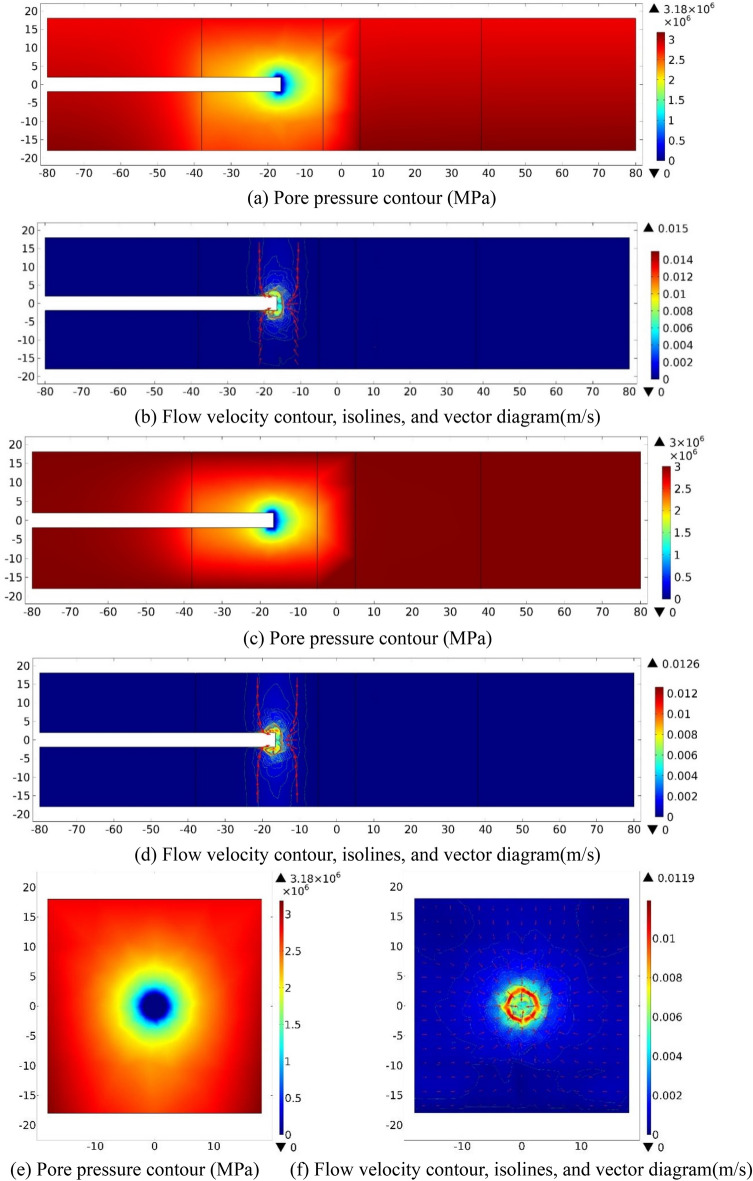
Numerical investigation results for the case *S* = −16.5 m. (**a**) and (**b**): XZ_Y=0_ section; (**c**) and (**d**): XY_Z=0_ section; (**e**) and (**f**): YZ_X=−16.5_ section.

While the tunnel face is excavated to the center of the fault center core of Fault 1 (S = −16.5), the water inflow numerical investigation results on three sections (XZ_Y=0_, XY_Z=0_, YZ_X=−16.5_) are shown in Fig. 7 as follow.

As displayed in the pore pressure contours, *P* increases quickly from a low value at the working face to a certain maximum value with the increases of *D*. On the section of XZ_Y=0_, *P* contour is elliptical that parallel to X-axis on the long axis and changes step by step to an approximate rectangular distribution. The low-pressure zone is significantly larger than that when the tunnel is excavated to *S* = −48 and *S* = −28. On the section of XY_Z=0_, the pore pressure gradient along the X-axis is greater than that along the Y-axis, and *P* far from the tunnel face is constant. On YZ_X=−16.5_ section, *P* is distributed in a circular distribution. The pressure gradually increases from the tunnel perimeter into the deeper foundation. The pressure distribution pattern becomes an oval distribution parallel to Z-axis on the long axis.

It can be seen from the flow velocity contours that the value of *V* within 1 m ahead of the excavation face is comparatively big, then decreasing, while the groundwater flows towards the tunnel. On XZ_Y=0_ section, *V* contour is axisymmetrically elliptical with respect to X-axis and parallel to Z-axis on the long axis, and the maximum value of *V* is 0.015 m/s. On the section of XY_Z=0_, *V* contour shows an oval distribution parallel to Y-axis on the long axis, and the maximum value of *V* is 0.0126 m/s. On YZ_X=−16.5_ section, the flow velocity contour shows a circular distribution, with a maximum of 0.0119 m/s. In the case of parallel faults, while the tunnel face is excavated into the center of the fault center core of Fault 1, groundwater flows into the tunnel. This is because pore pressure at and 1 m behind the excavation face is dissipated, forming a low-pressure area. The decrease in pore pressure leads to an increase in hydraulic gradient, and the groundwater is more likely to flow into the vicinity of the tunnel face.

(2) Analysis and summary

**Figure 8:**
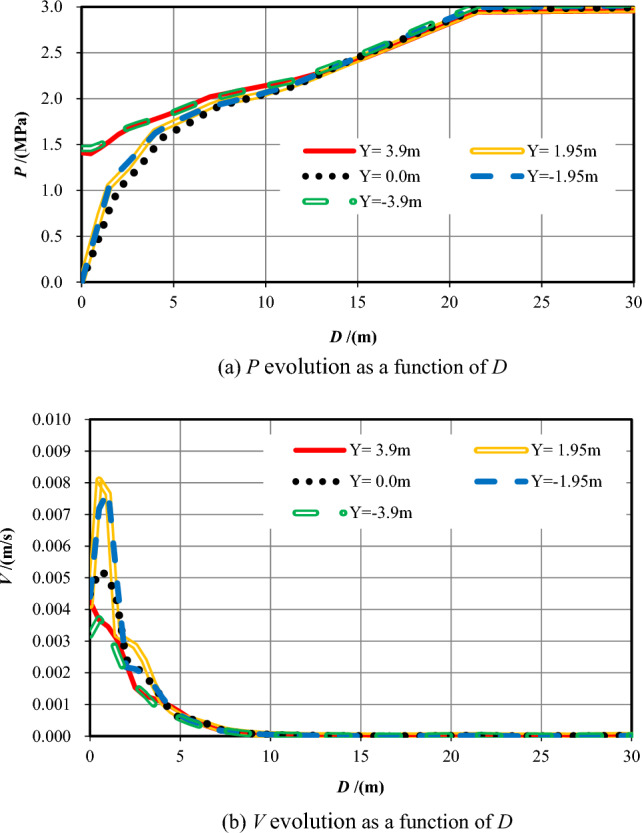
Pore pressure and flow velocity within 30 m ahead of the excavation face (*S* = −16.5).

Table 4 reveals the information of the survey range and the measuring points number while *S* = −16.5. Figure 8 shows the evolution data of *P* and *V* that monitored within 30 m ahead of the excavation face as *D*.

**Table 4:** Information of the measuring points while *S* = *−*16.5.

No. of measuring line	Survey range	Number
11	X = *−*16.5–33.5 m, Y = 3.90 m	100
12	X = *−*16.5–33.5 m, Y = 1.95 m	100
13	X = *−*16.5–33.5 m, Y = 0.00 m	100
14	X = *−*16.5–33.5 m, Y = *−*1.95 m	100
15	X = *−*16.5–33.5 m, Y = *−*3.90 m	100

Figure 8a shows that while Y =  ± 3.9 m, the similar pore pressures because of equal distance from the excavation face are 1.41 MPa and 1.46 MPa, separately. The *P* value increases as *D* increases (0 < *D* ≤ 10 m) and eventually reaches to 3 MPa. While Y =  ± 1.95 m and 0, the pore pressure values at the working face are all 0. While 0 < *D* ≤ 5 m, the pore pressure increases quickly and then slowly from 0 MPa at and within 5 m in front of the working face, eventually stabilizing at around 3 MPa when *D* > 5 m. While 0 < *D* ≤ 5 m, the *P* values when Y =  ± 3.9 m are bigger than that when Y =  ± 1.95 m and 0.

Figure 8b reveals that while Y =  ± 3.9 m, *V* declines quickly ahead (0 < *D* ≤ 6 m) and then decreases slowly into the deep formation (6 < *D* ≤ 10 m), afterwards, it approaches to 0. *V*_*max*_ appears at and within 0.5 m ahead of the excavation face, that are 0.0042 m/s and 0.0037 m/s, separately. When Y =  ± 1.95 m and 0, *V* increases quickly within 0.5–1 m first , and then declines rapidly ahead (0 < *D* ≤ 6 m). The *V*_*max*_ values appear at *D* = 0.5 m, 0.5 m, and 1.0 m, respectively, which are 0.0081 m/s, 0.0052 m/s, and 0.0077 m/s. While 0 < *D* ≤ 5 m, the values of *V* when Y = 1.95 m, 0 and −1.95 m are much bigger than that when Y =  ± 3.9 m. While *D* > 5 m into the deep formation, they are almost equal.

### Tunnel excavated to the overlapping center of the parallel faults (S = 0)

(1) Numerical investigation results

**Figure 9:**
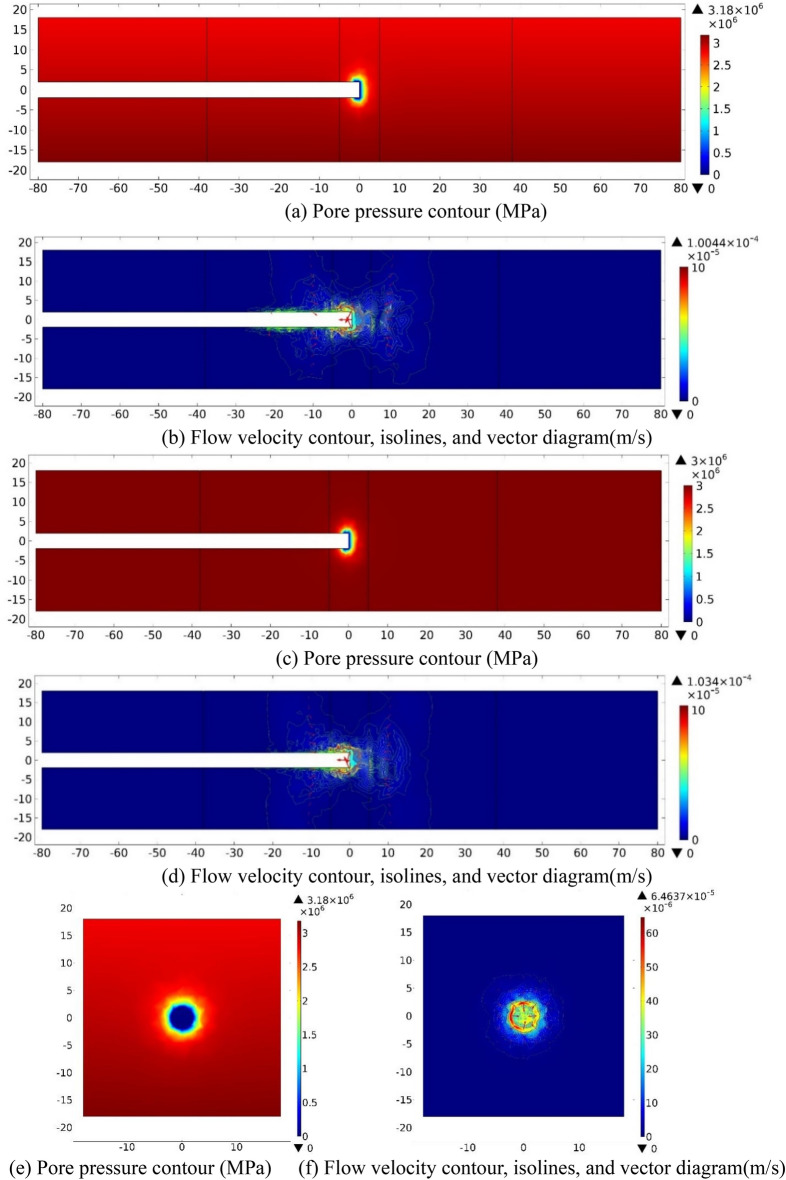
Numerical investigation results for the case *S* = 0. (**a**) and (**b**): XZ_Y=0_ section; (**c**) and (d): XY_Z=0_ section; (**e**) and (**f**): YZ_X=0_ section.

While the tunnel face reached to the center of overlapping zone of the parallel faults (S = 0), the water inflow numerical investigation results on three sections (XZ_Y=0_, XY_Z=0_, YZ_X=0_) are shown in Fig. 9 as follow.

As plotted in Fig. 9a,c,e, in the fault center core and the fractured zone of fault, a low-pressure area appears at about 1 m range behind the tunnel excavation face. On the *S* = 0 tunneling position, the low-pressure area is much smaller than the areas on *S* = −48 m, −28 m, and −16.5 m. On XZ_Y=0_ and XY_Z=0_ sections, pore pressure presents an oval distribution with the major axis parallel to X-axis. On YZ_X=0_ section, the pressure distribution is approximately circular. Flow velocity contours show that *the value of V* within 1 m ahead of the excavation face appears big, then decreasing to 0. The flow velocity gradient along the Z-axis is greater than that along the X-axis, with the maximum *V* value of 10 × 10^−5^ m/s. On YZ_X=0_ section, the maximum value of *V* is 6.46 × 10^−5^ m/s.

*P* contours show a low-pressure zone around the excavation face at the condition of *S* = 0. Since the permeability of the overlapping area is the superposition of the permeability of the two damage zones, which is higher than that of a single damage zone. The pore pressure Adjacent to the overlapped area of the two faults decreases rapidly. As a result, *the value of V* in the fault center core is much bigger than that in the ordinary rock zone and reaches its maximum value at the vicinity of the tunnel excavation face.

(2) Analysis and summary

**Figure 10:**
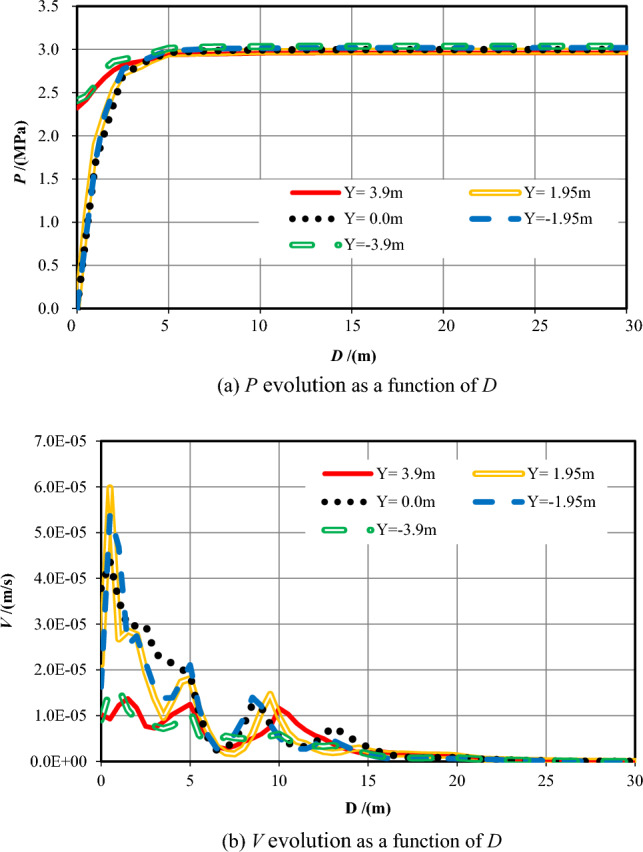
*P* and *V* evolution within 30 m ahead of the tunnel face (*S* = 0).

Similarly, Table 5 reveals the information of the survey range and the measuring points number while *S* = −28. Figure 10 shows the evolution data of *P* and *V* monitored within 30 m ahead of the excavation face as *D*.

**Table 5:** Information of the measuring points while *S* = 0.

No. of measuring line	Survey range	Number
16	X = 0–50 m, Y = 3.90 m	100
17	X = 0–50 m, Y = 1.95 m	100
18	X = 0–50 m, Y = 0.00 m	100
19	X = 0–50 m, Y = *−*1.95 m	100
20	X = 0–50 m, Y = *−*3.90 m	100

Figure 10a reveals that while Y =  ± 3.9 m, the *P* values at the excavation face are around 2.3–2.4 MPa, then increases as the increase of *D* (0 < *D* ≤ 5 m) and eventually reaches to 3 MPa within the deep formation. *P* increases quickly first and then slowly from 0 MPa at and within 3–5 m in front of the working face, eventually stabilizing at around 3 MPa. While 0 < *D* ≤ 5 m, the *P* values when Y =  ± 3.9 m are bigger than that when Y =  ± 1.95 m and 0.

Figure 10b reveals that while Y =  ± 3.9 m, *V* declines quickly ahead (0 < *D* ≤ 15 m) and then decreases slowly into the deep formation, afterwards, it approaches to 0. The flow velocity fluctuates in the 1–2 m, 3–5 m, and 7–10 m sections in front of the working face. The maximum values of *V* occur at *D* = 1.5 m and *D* = 1 m. After that, flow velocity changes slightly deeper into the formation, eventually reaching to zero. When Y =  ± 1.95 m and 0, *V* generally decreases in the 15 m range ahead of the excavation face. While *D* is within 0.5 m ahead, *V* rapidly increases. Then, the flow velocities fluctuate in the ranges of 3.5–7.5 m, 7.5–15 m, and *V*_*max*_ occur at *D* = 5.5 m and 9.5 m, respectively. Within 0.5–7 m range, flow velocity on the Y = −1.95 m section decreases rapidly. Within 7–11 m and 11–15 m, flow velocities increase and then decrease, reaching peaks at *D* = 8.5 m and 13 m, respectively. While *D* > 15 m, *V* slowly decreases deeper into the formation. While 0 < *D* ≤ 3 m, the values of *V* when Y = 1.95 m, 0 and −1.95 m are much bigger than that when Y =  ± 3.9 m. Outside of the 10 m area, the difference is small.

### Tunnel excavated to 16.5 m ahead the center of parallel faults (S = 16.5)

(1) Numerical investigation results

**Figure 11:**
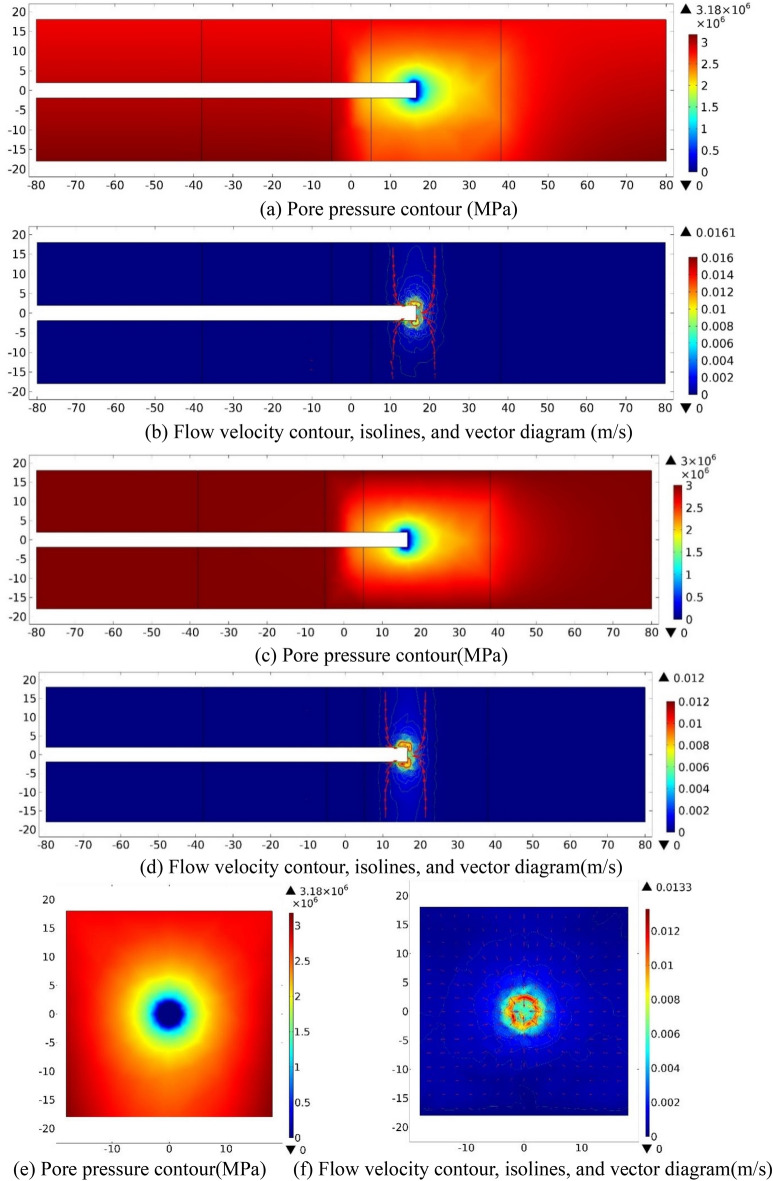
Numerical investigation results for the case *S* = 16.5 m. (**a**) and (**b**): XZ_Y=0_ section; (**c**) and (**d**): XY_Z=0_ section; (**e**) and (**f**): YZ_X=16.5_ section.

While the tunnel face is excavated to the center of the fault center core of Fault 2 (S= 16.5), the water inflow numerical investigation results on three sections (XZ_Y=0_, XY_Z=0_, YZ_X=−16.5_) are shown in Fig. 11 as follow.

Figure 11 reveals that the distribution of *P* when *S* = 16.5 m is equivalent to that when *S* = −16.5 m. Groundwater flows into the tunnel, and the pressure around and at the perimeter 1 m behind the excavation face wears off, taking a distribution of low-pressure area. The decrease in pore pressure causes the hydraulic gradient to increase, hence groundwater flows into the vicinity of the working face. Since the permeability value of the wall rock in the fault center core is much bigger than that of the fractured zones, the value of *P* decreases rapidly, the direction of *V* is from the two sides of the fault center core of Fault 2 to low-pressure zone. *V* values of the groundwater in fault zone are larger than that in the ordinary rock zones and is the highest Adjacent to the tunnel face.

(2) Analysis and summary

**Figure 12:**
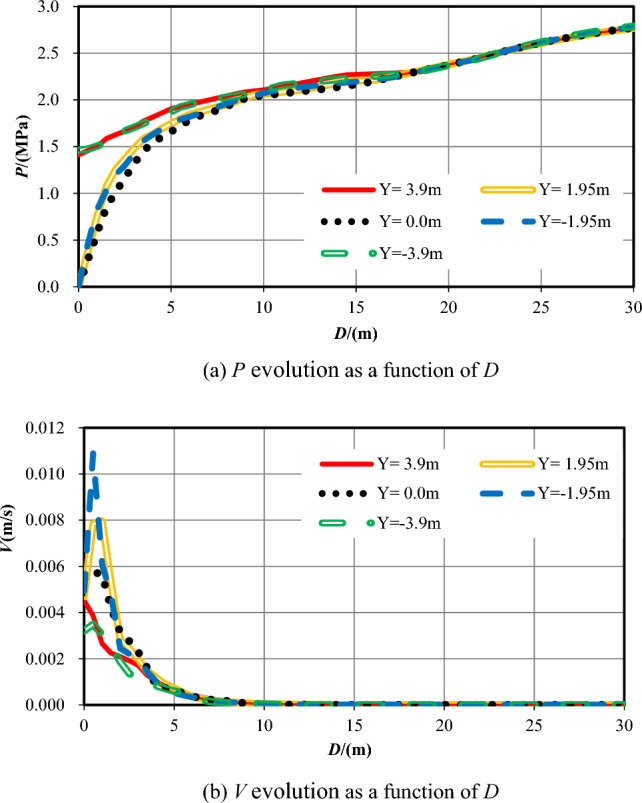
Pore pressure and flow velocity within 30 m ahead of the excavation face (*S* = 16.5 m).

Again, Table 6 reveals the information of the survey range and the measuring points number while *S* = −28. Figure 12 shows the evolution data of *P* and *V* that monitored within 30 m ahead of the excavation face as *D*.

**Table 6:** Information of the measuring points while *S* = 16.5.

No. of measuring line	Survey range	Number
21	X = 16.5–66.5 m, Y = 3.90 m	100
22	X = 16.5–66.5 m, Y = 1.95 m	100
23	X = 16.5–66.5 m, Y = 0.00 m	100
24	X = 16.5–66.5 m, Y = *−*1.95 m	100
25	X = 16.5–66.5 m, Y = *−*3.90 m	100

Figure 12a shows that while the tunnel face is excavated into the fault center core center of Fault 2, the value of *P* at and around the range of 1 m behind the excavation face is zero, then rapidly increase in the fractured zone, and then slowly increases. The value of *P* along the Y-axis within the range of −1.95 m ≤ Y ≤ 1.95 m is significantly lower than that in other areas, and the difference in pore pressure away from the tunnel face is not apparent. Figure 12b reveals that the maximum value of *V* appears at the excavation face. The flow velocity value of groundwater is high around the excavation face, decreases rapidly forward, and changes gently in the ordinary rock zone. Along the Y-axis, the value of *V* at the excavation face is much bigger than that on both sides. Into the deeper formation that away from the excavation area, the flow velocity on each measuring line changes insignificantly, close to 0.

### Excavated face is 28 m ahead the center of parallel faults (S = 28)

(1) Numerical investigation results

**Figure 13:**
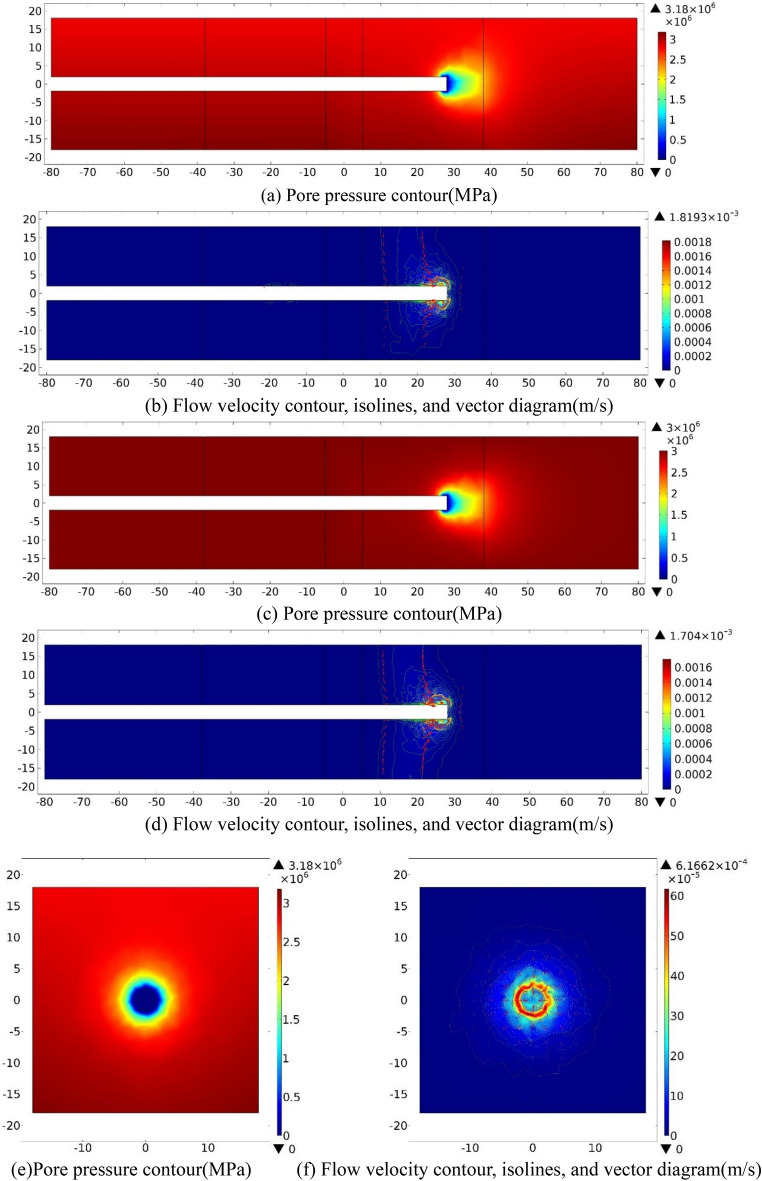
Numerical investigation results for the case *S* = 28 m. (**a**) and (**b**): XZ_Y=0_ section; (**c**) and (**d**): XY_Z=0_ section; (**e**) and (**f**): YZ_X=28_ section.

While the tunnel excavation face arrives at the center of the right fractured zone of Fault 2 (*S* = 28), the water inflow numerical simulation results on three sections (XZ_Y=0_, XY_Z=0_, YZ_X=−28_) are shown in Fig. 13 as follow.

As plotted in Fig. 13a,c,e, a low-pressure area appears around and 1.0 m range behind the excavation face in the fractured zone of Fault 2, which is similar to the situation on the Y = −28 m section. Groundwater flows toward the tunnel face area. As *V* contours shown in Fig. 13b,d,f, the distribution law of *V* of the groundwater is not similar as other cases, and it is larger within 1 m of the tunnel perimeter and gradually decreases. The flow direction of groundwater is toward the excavation face. On section of XZ_Y=0_, the flow velocity contour is in an arc distribution, and gradually offsets to the area between the directions of the negative X-axis and the positive Z-axis, with the maximum *V* value of 1.82 × 10^−3^ m/s. On section of YZ_X=28_, *V* contour of the groundwater is in an approximately circular distribution, and with the maximum value is 6.17 × 10^−4^ m/s. Since the permeability in the fractured zone of Fault 2 is higher than that in the ordinary rock zone, but lower than that of the fault center core, groundwater flows from the two sides of the fault center to the tunnel. The velocity value of the groundwater in the fault center core area is also bigger than that in the fractured zone and host rock. The highest velocity appears at the tunnel perimeter that about 1 m away behind the excavation face.

(2) Analysis and summary

**Figure 14:**
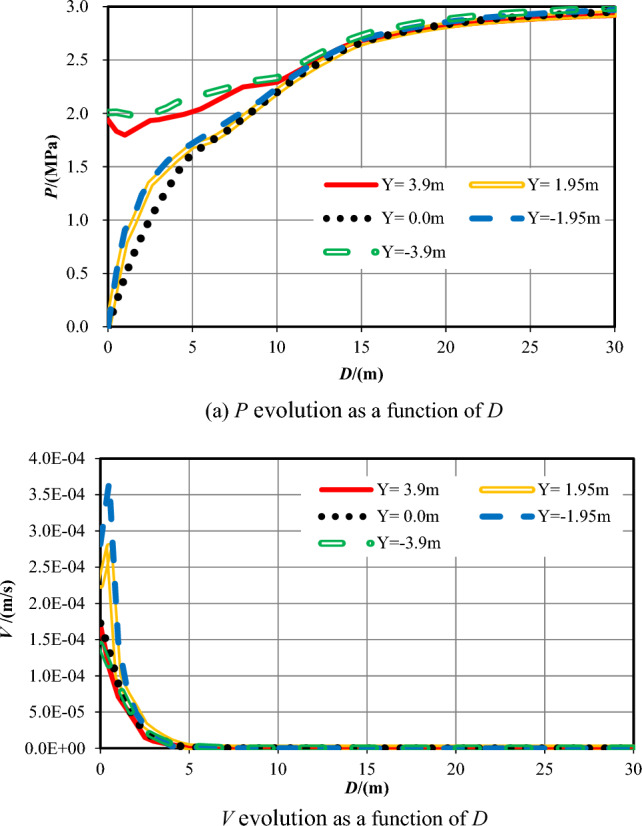
*P* and *V* ahead of the tunnel face (*S* = 28 m).

Table 7 reveals the information of the survey range and the measuring points number with the case of *S* = 28. Figure 14 reveals the evolution data of *P* and *V* of groundwater that monitored within 30 m ahead of the excavation face as *D*.

**Table 7:** Information of the measuring points while *S* = 28.

No. of measuring line	Survey range	Number
26	X = 28–78 m, Y = 3.90 m	100
27	X = 28–78 m, Y = 1.95 m	100
28	X = 28–78 m, Y = 0.00 m	100
29	X = 28–78 m, Y = *−*1.95 m	100
30	X = 28–78 m, Y = *−*3.90 m	100

Figure 14a shows that while the excavation face is excavated into the center of the fractured zone of Fault 2, the value of *V* grows quickly at the very beginning and then tardily to 3.0 MPa. Along the direction of Y-axis, the value of *P* in the vicinity of the excavation face is much smaller than in other areas. Water pressure far from the excavation face decreases when the value of Y increases. Figure 14b reveals that the maximum value of *V* appears at the vicinity of the excavation face of tunnel. The value of *V* is high adjacent to the tunnel face, decreases rapidly forward, and then changes slowly. Along the direction of Y-axis, the value of *V* in the working face is obviously bigger than that in the area on both sides. Far away from the excavation face, the value of *V* changes little and is close to zero.

### Excavated face is 48 m ahead the center of parallel faults (S = 48)

(1) Numerical investigation results

**Figure 15:**
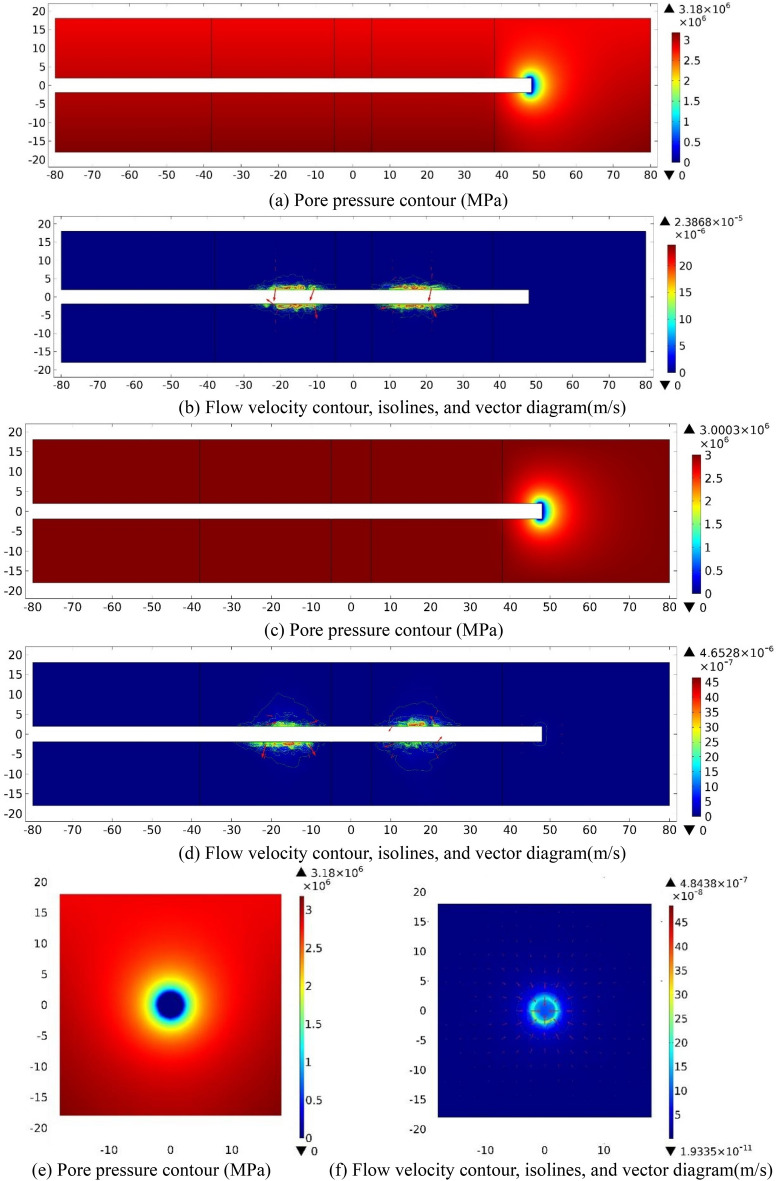
Numerical investigation results for the case *S* = 48 m. (**a**) and (**b**): XZ_Y=0_ section; (**c**) and (**d**): XY_Z=0_ section; (**e**) and (**f**): YZ_X=48_ section.

When the tunnel is excavated to the section that 10 m away from the right boundary of the fractured zone of Fault 2(*S* = 48), the water inflow numerical simulation results on three sections (XZ_Y=0_, XY_Z=0_, YZ_X=−48_) are shown in Fig. 15 as follow.

As shown in *P* contours, a low-pressure area is presented within 1 m range of the excavation face in the fault center core and fractured zone which is similar with the cases of *S* = −48 m. Particularly, the value of *P* equals to 0 at the excavation face and grows quickly as *D* increases. On XZ_Y=0_ and XY_Z=0_ sections, pore pressure gradients along the X-axis are smaller than those along other directions. Pore pressure shows an constant value in the deep wall rock that far from the excavation face. On the section of YZ_X=0_, *P* contour is nearly circular adjacent to the tunnel perimeter. Contours and vector diagrams of *V* reveal that the magnitude of *V* value in the model is only 10^−7^ m/s, while the maximum value of *V* occurs at the excavation face and the perimeter nearby. On XZ_Y=0_ and XY_Z=0_ sections, the contour of *V* is approximately circular and symmetric in the X-axis, and the maximum values of *V* are 2.39 × 10^−5^ m/s and 4.65 × 10^−6^ m/s, separately. On YZ_X=0_ section, the contour of *V* also shows a circular distribution and with a maximum value of about 4.84 × 10^−7^ m/s.

(2) Analysis and summary

**Figure 16:**
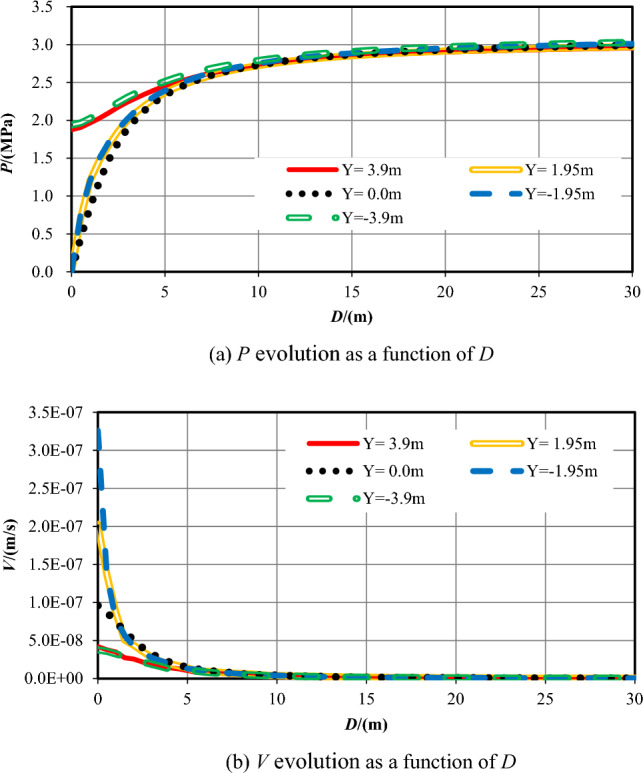
Pore pressure and flow velocity within 30 m ahead of the tunnel face (*S* = 48 m).

Table 8 reveals the information of the survey range and the measuring points number while *S* = 48. Figure 16 shows the groundwater evolution data of *P* and *V* that monitored within 30 m ahead as a function of *D*.

**Table 8:** Information of the measuring points while *S* = 48.

No. of measuring line	Survey range	Number
31	X = 48–78 m, Y = 3.90 m	100
32	X = 48–78 m, Y = 1.95 m	100
33	X = 48–78 m, Y = 0.00 m	100
34	X = 48–78 m, Y = *−*1.95 m	100
35	X = 48–78 m, Y = *−*3.90 m	100

On Y =  ± 3.9 m sections, Fig. 16a shows that the values of *P* at the excavation face are 1.88 MPa and 1.94 MPa, separately. While 0 ≤ *D* ≤ 6 m, *P* grows rapidly ahead with the increase of *D* value, and eventually stabilize at the maximum value. While Y =  ± 1.95 m and 0, the value of *P* at the excavation face are approximately zero. While 0 ≤ *D* ≤ 6 m, the value of *P* increases quickly, then it rises gradually and afterward stabilized at the maximum value. The value of *P* on sections of Y =  ± 3.9 m is larger than that on the sections of Y = 1.95 m, 0 and −1.95 m in the range of 0 ≤ *D* ≤ 6 m, while the value of *P* on the section of Y = −3.9 m is slightly larger than that on the section of Y = 3.9 m. Figure 16b shows that the flow velocity drops rapidly within 6 m in front of the excavation face and then gradually descends, and the maximum values of *V* all appear at the tunnel excavation face. While 0 ≤ *D* ≤ 6 m, the values of *V* on the sections of Y =  ± 1.95 m and 0 are higher than that on the section of Y =  ± 3.9 m, and the maximum appears on the Y = −1.95 m section. Outside the 6 m range, the flow velocity tends to 0.

## Discussion

**Figure 17:**
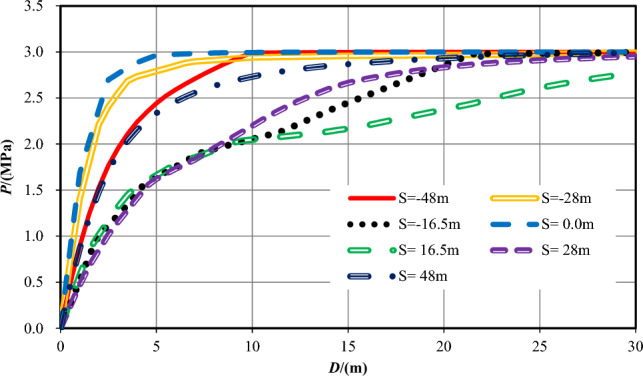
*P* evolution as a function of *D* for seven different *S* cases.

**Figure 18:**
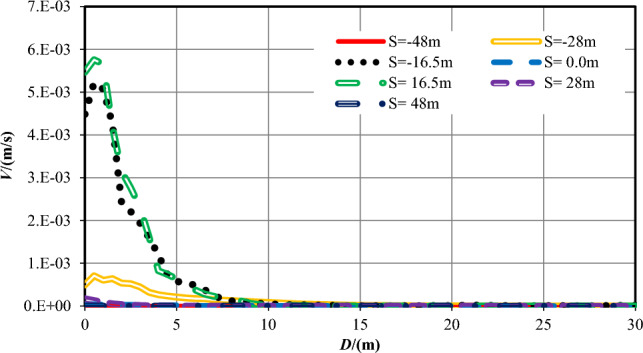
V evolution as a function of *D* for seven different *S* cases.

In the earlier sections of this work, the numerical calculation results and analysis of the pore pressure and flow velocity contours are outlined while the tunnel excavation face arrives at seven typical positions of two overlapped parallel faults, while the strike of the fault is vertical and perpendicular to the center axis of the tunnel. In the sections that follow, combining with these analysis results, the influence of the relative position and distance between the tunnel excavation face and the two overlapped parallel faults on water inflow into tunnel is focused. Figures 17 and 18 reveal that all the pore pressure curves and the flow velocity curves for seven varies cases at the measuring points at the central line of the tunnel (Y = 0 m).

While the tunnel face is excavated to the centers of the fault center cores, which is *S* =  ± 16.5 m, the permeability of the wall rock is the largest. The increase velocity of the pore pressures ahead of the excavation face is the lowest, so the range affected by the excavation and support is the biggest. While the tunnel excavation face is in the centers of fractured zones (*S* =  ± 28 m), the permeability of the rock is reduced, the increase velocity of the pore pressure ahead of the excavation face a little big, and the range influenced by the construction is diminished. While the tunnel is excavated into the ordinary rock zones (*S* =  ± 48 m), the permeability of the wall rock is the smallest, and the pore pressure values ahead of the excavation face increases quickly. When the tunnel excavation face reaches into the center of the overlapped area (*S* = 0 m), the permeability in front of the excavation face rises the fastest.

While the tunnel is excavated to the center of the fault cores, which is *S* =  ± 16.5 m, the flow velocity values at the excavation face is also the biggest because of the biggest permeability value of the wall rock, significantly higher than that under other working conditions. In the centers of damage zones (*S* =  ± 28 m), the permeability of the rock is secondary, and the values of flow velocity at the excavation face is also reduced. In the center of the two parallel overlapped faults (*S* = 0 m), the permeability of this area is greater than that of the ordinary rock zone but smaller than that of the fractured zone. Therefore, the value of flow velocity here is lower than that in the fractured zone. When the tunnel excavation face is in the ordinary rock zones (*S* =  ± 48 m), the permeability of the rock is the smallest, and the value of *V* around the excavation face is also the lowest. With two parallel faults, the water inflow rate into tunnel on the excavation face, at the tunnel perimeter and the total are shown in Table 9 and Fig. 18.

**Table 9:** Water inflow rates at different sections in parallel faults conditions.

Calculation conditions	*S* = *−*48	*S* = *−*28	*S* = *−*16.5	*S* = 0	*S* = 16.5	*S* = 28	*S* = 48
Water inflow rate on the excavation face /(m^3^/h)	0.0055	19.890	238.990	1.390	243.830	8.800	0.0053
Water inflow rate on the tunnel perimeter /(m^3^/h)	0.0150	35.510	558.720	2.870	630.070	33.140	0.0150
Total /(m^3^/h)	0.0205	55.400	797.710	4.260	873.900	41.940	0.0203

**Figure 19:**
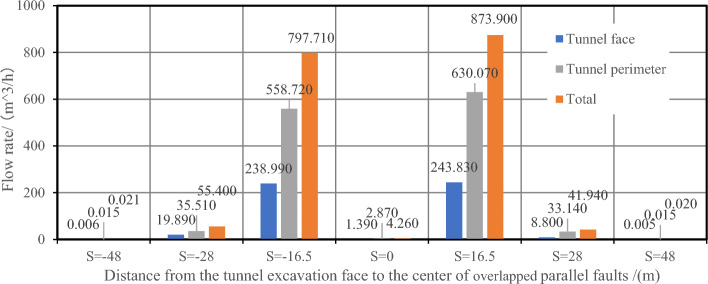
Water inflow rate vs. distance from the excavation face to the center of the overlapped parallel faults.

It can be seen from Table 9 and Fig. 19 that in the case of two parallel faults with vertical directions, when the excavation face reaches into the centers of the fault center cores (*S* =  ± 16.5 m), the water inflow rate is the largest. It is because the fault center core has the highest permeability, and the fluid can flow into the tunnel in the largest amount per unit of time. When the tunnel face is excavated into the enters the fractured zone (*S* =  ± 28 m), the water inflow rate ranks the second. The reason is that the permeability of the fractured zone is between the biggest that of the fault center core and the smallest that of ordinary rock zone. While the tunnel is excavated into the center of the overlapped parallel faults (*S* = 0 m), the water inflow rate is less than that in the fractured zones (*S* =  ± 28 m). Although this area has the superposition of the permeability of the two faults zones, the permeability in the fractured zone conforms to a Gaussian function distribution. Hence, it is higher than that of the ordinary rock zone but lower than that of the fractured zone. In addition, the value of water inflow rate in the fractured zone is closely related to the excavation position of the excavation face. The smaller the relative distance away from the excavation face to the fault center core, the greater the inflow rate. The permeability of the ordinary rock zone in the non-faulted area is the smallest, where the water inflow rate is also the smallest. When the tunnel crosses two closely spaced parallel faults, the water inflow rate is the largest in the center of the fault center core. Therefore, more attention should be paid in these areas and preventive and drainage measures should be taken as well.

## Conclusions

The effect of the relative distance away from the excavation face to the center of the overlapped parallel faults to the evolution law of the pore pressure, the flow velocity, and the water inflow into tunnel was evaluated by solving the improved Darcy-Brickman equation, when a tunnel passes through two overlapped parallel faults perpendicularly. The conclusions are drawn as follow.

1. Pore pressure. While the tunnel excavation face reaches to the fault center core, the pore pressure value ahead of the excavation face increases with the slowest velocity, and the range affected by the excavation is the largest. While the tunnel excavation face arrives at the center of the fractured zone, the pore pressure value ahead increases with a slightly faster velocity, and the range affected by the excavation decreases. While the working face reaches the ordinary rock zone, the pore pressure value ahead increases quickly. Finally, while the excavation face reaches to the center of the overlapped area of the two parallel faults, pore pressure increases the fastest and has the smallest influence area.
2. Flow velocity. While the excavation face reaches to the fault center core, the flow velocity value on the excavation face is much bigger than that at other locations. While the tunnel excavation face is in the center of the fractured zone, the flow velocity value near by the excavation face is relatively lower. While the excavation face arrives at the center of the overlapped fault area, the value is lower than that in the fractured zone.
3. Inflow rate. While the tunnel excavation face reaches to the fault affecting zone, the water inflow rate is greatly influenced by the relative location of the tunnel excavation face and the fault center core. The smaller the distance away from the tunnel excavation face to the fault core, the higher the water inflow rate. Conversely, the water flow velocity is relatively small when the tunnel is out of faulted areas.

## Data Availability

The data that support the findings of this study are available from the corresponding author upon reasonable request.

## References

[1] Gong H (2023). Determination of the damage degree of coal rocks and study on the evolution law of permeability based on Zhaogu No. 2 Coal Mine. J. Min. Strata Control Eng..

[2] Li S (2015). Detecting and monitoring of water inrush in tunnels and coal mines using direct current resistivity method: A review. J. Rock Mech. Geotech. Eng..

[3] Xu Z (2021). Hydro-mechanical coupling response behaviors in tunnel subjected to a water-filled karst cave. Rock Mech. Rock Eng..

[4] Wang Y (2012). Risk assessment of floor water inrush in coal mines based on secondary fuzzy comprehensive evaluation. Int. J. Rock Mech. Min. Sci..

[5] Li SC (2021). Numerical investigation of hydraulic tomography for mapping karst conduits and its connectivity. Eng. Geol..

[6] Wang XT (2019). An interval risk assessment method and management of water inflow and inrush in course of karst tunnel excavation. Tunn. Undergr. Space Technol..

[7] Li X (2017). Identification of geological structure which induced heavy water and mud inrush in tunnel excavation: a case study on Lingjiao tunnel. Tunn. Undergr. Space Technol..

[8] Wang XT (2019). Risk assessment of water inrush in karst tunnels excavation based on normal cloud model. Bull. Eng. Geol. Env..

[9] Sun W (2016). Hydrogeological classification and water inrush accidents in China's coal mines. Mine Water Environ..

[10] Guo JQ (2018). Water Inrush Criterion and Catastrophe Process of a Karst Tunnel Face with Non-persistent Joints. China J. Highway Transp..

[11] Li L (2016). Mechanism of water inrush in tunnel construction in karst area. Geomat. Nat. Haz. Risk.

[12] Wang Y (2012). Fault-dominated water model and multi-factor method for predicting water inflow and inrush of deep long tunnel in fractured rock masses. J. Rock Mech. Geotech. Eng..

[13] Xue YG (2021). Water and mud inrush hazard in underground engineering: Genesis, evolution and prevention. Tunn. Undergr. Space Technol..

[14] Xu ZH (2022). A novel numerical method for grouting simulation in flowing water considering uneven spatial and temporal distribution of slurry: Two-Fluid Tracking (TFT) method. Comput. Geotech..

[15] Wang SM (2020). Scientific issues of coal detraction mining geological assurance and their technology expectations in ecologically fragile mining areas of Western China. J. Min. Strata Control Eng..

[16] Li SC (2017). Gaussian process model of water inflow prediction in tunnel construction and its engineering applications. Tunn. Undergr. Space Technol..

[17] Zheng Z (2019). Numerical simulation and risk assessment of water inrush in a fault zone that contains a soft infill. Mine Water Environ.

[18] Jeon S (2004). effect of a fault and weak plane on the stability of a tunnel in rock-a scaled model test and numerical analysis. Int. J. Rock Mech. Min. Sci..

[19] Wang J (2021). Seepage characteristic and fracture development of protected seam caused by mining protecting strata. J. Min. Strata Control Eng..

[20] Farhadian H, Nikvar-Hassani A (2019). Water flow into tunnels in discontinuous rock: a short critical review of the analytical solution of the art. Bull. Eng. Geol. Env..

[21] Moon J, Jeong S (2011). Effect of highly pervious geological features on ground-water flow into a tunnel. Eng. Geol..

[22] Tang S (2018). Numerical modeling of the time-dependent development of the damaged zone around a tunnel under high humidity conditions. Tunn. Undergr. Space Technol..

[23] Butscher C, Scheidler S, Farhadian H, Dresmann H, Huggenberger P (2017). Swelling potential of clay-sulfate rocks in tunneling in complex geological settings and impact of hydraulic measures assessed by 3D groundwater modeling. Eng. Geol..

[24] Gao C (2021). Peridynamics simulation of water inrush channels evolution process due to rock mass progressive failure in karst tunnels. Int. J. Geomech..

[25] Zhang P, Xu D, Fu X (2022). Evaluation of hydraulic conductivity based on fault confinement studies. Journal of Mining and Strata Control Engineering.

[26] Wu J, Wu L, Sun M (2022). Analysis and research on blasting network delay of deep-buried diversion tunnel crossing fault zone based on EP-CEEMDAN-INHT. Geotech. Geol. Eng..

